# Antecedent use of renin-angiotensin system inhibitors is associated with reduced mortality in elderly hypertensive Covid-19 patients

**DOI:** 10.1097/HJH.0000000000003059

**Published:** 2021-12-09

**Authors:** Mauro Gori, Carlo Berzuini, Emilia D’Elia, Arianna Ghirardi, Luisa Bernardinelli, Antonello Gavazzi, Giulio Balestrieri, Andrea Giammarresi, Roberto Trevisan, Fabiano Di Marco, Antonio Bellasi, Mariangela Amoroso, Federico Raimondi, Luca Novelli, Bianca Magro, Gianpaolo Mangia, Ferdinando L. Lorini, Giulio Guagliumi, Stefano Fagiuoli, Gianfranco Parati, Michele Senni

**Affiliations:** a Cardiovascular Department, Bergamo, Italy; b The Unit of Pulmonary Medicine, Bergamo, Italy; c The Gastroenterology Hepatology and Transplantation Unit, Bergamo, Italy; d The Intensive Care Unit, Bergamo, Italy; e FROM Research Foundation, Bergamo, Italy; f The Endocrinology Unit, Bergamo, Italy; g The Department of Research, Innovation, Brand Reputation, Bergamo, Italy; h ASST Papa Giovanni XXIII, Bergamo, Italy; i Centre for Biostatistics, School of Health Sciences, The University of Manchester, Manchester, UK; j The Department of Brain and Behavioural Sciences, The University of Pavia, Pavia, Italy; k Istituto Auxologico Italiano, IRCCS, Department of Cardiovascular, Neural and Metabolic Sciences, S. Luca Hospital; l University of Milan; m Department of Medicine and Surgery, University of Milan-Bicocca, Milan, Italy

**Keywords:** angiotensin receptor blockers, angiotensin-converting enzyme inhibitors, Covid-19, elderly, hypertension, observational study, propensity score matching, renin-angiotensin-system inhibitors

## Abstract

**Objectives:**

The effect of renin-angiotensin system inhibitors (RASIs) on mortality in patients with coronavirus disease (Covid-19) is debated. From a cohort of 1352 consecutive patients admitted with Covid-19 to Papa Giovanni XXIII Hospital in Bergamo, Italy, between February and April 2020, we selected and studied hypertensive patients to assess whether antecedent (prior to hospitalization) use of RASIs might affect mortality from Covid-19 according to age.

**Methods and results:**

Arterial hypertension was present in 688 patients. Overall mortality (in-hospital or shortly after discharge) was 35% (*N* = 240). After adjusting for 26 medical history variables via propensity score matching, antecedent use of RASIs (*N* = 459, 67%) was associated with a lower mortality in older hypertensive patients (age above the median of 68 years in the whole series), whereas no evidence of a significant effect was found in the younger group of the same population (*P* interaction = 0.001). In an analysis of the subgroup of 432 hypertensive patients older than 68 years, we considered two RASI drug subclasses, angiotensin-converting enzyme inhibitors (ACEIs, *N* = 156) and angiotensin receptor blockers (ARBs, *N* = 140), and assessed their respective effects by taking no-antecedent-use of RASIs as reference. This analysis showed that both antecedent use of ACEIs and antecedent use of ARBs were associated with a lower Covid-19 mortality (odds ratio_ACEI_ = 0.57, 95% confidence interval 0.36--0.91, *P* = 0.018) (odds ratio_ARB_ = 0.49, 95% confidence interval 0.29--0.82, *P* = 0.006).

**Conclusion:**

In the population of over-68 hypertensive Covid-19 patients, antecedent use of ACEIs or ARBs was associated with a lower all-cause mortality, whether in-hospital or shortly after discharge, compared with no-antecedent-use of RASIs.

## INTRODUCTION

Over the past months, a number of articles have addressed the impact of commonly used anti-hypertensive drugs, such as renin-angiotensin system (RAS) inhibitor drugs (RASIs) and, in particular, angiotensin-converting enzyme inhibitors (ACEIs) and angiotensin receptor blockers (ARBs), on the consequences of coronavirus disease (Covid-19), often reaching different conclusions. A possible adverse effect of RASIs in Covid-19 patients is suggested by evidence that the ACE2 (a negative regulator of RAS) acts as a receptor for the Covid-responsible virus (SARS-Cov-2) to enter the infected cells and replicate [1]. Evidence from animal studies also suggests that certain RASIs might upregulate ACE2 [2,3] and, as a consequence, help SARS-Cov-2 invade human cells.

Given that RASIs are widely used in the most Covid-19-vulnerable part of the population, that is the elderly, the prospect of a Covid-19-vulnerable population making widespread use of drugs suspected to worsen that disease has raised deep concern. These fears have been tempered by epidemiological studies showing no evidence of whatsoever effect of RASIs on Covid-19 outcomes [2,4–8]. In most cases, these studies investigated the effect of antecedent use of RASIs in hypertensive Covid-19 patients, concluding for a neutral effect. Their inability to provide significant evidence for a beneficial effect of these drugs might have different possible explanations, such as bias by comparing treated patients with patients not receiving any therapy [2], or the choice of receiving Covid-19 associated ICU admission (rather than a mortality) outcome [8]. Other studies rely on administrative data with partly incomplete information on clinically relevant data, such as comorbidities and drug use [4,5]. The size of the patient cohort in two Chinese studies was not sufficient to separately investigate ACEI and ARB treatments, which may have different mechanisms of action, while the number of sample patients on ACEI/ARB treatment was lower than expected, suggesting presence of unmeasured confounding [6,7]. Two studies claim a protective role of RASIs in hospitalized Covid-19 patients, but suffer from methodological flaws, such as adjustment for few variables, low statistical power, the inclusion of normotensive patients, which raises problems of comparability, and the lack of adjustment for age on a continuous scale within each broader age stratum [9,10]. On such a background, a firm conclusion about role of RASIs in affecting outcome of Covid-19 does not appear to have been reached, yet.

In such a context, it is worth noting that an international randomized clinical trial (RCT) on the effect of RASIs in Covid-19 (ClinicalTrials.gov Identifier: NCT04591210) will evaluate the potential benefits of angiotensin modulators on clinical outcomes, in older Covid-19 patients who are at high-risk for cardiovascular disease, in terms of mortality, ICU admission rate and ventilator requirement. Estimated study completion date is August 2022. Existence of these trials reflects the considerable interest of the medical community for the topic addressed in our article.

Aim of our study was indeed to clarify the effect on mortality of antecedent use of RASIs in Covid-19 patients with arterial hypertension. This was done by analysing data from a sample of consecutive hypertensive Covid-19 patients admitted to a single hospital, the Papa Giovanni XXIII Hospital in Bergamo, northern Italy, from 23 February to 7 April 2020. Importantly, we have explored the interaction between the effect of RASIs exposure and age, allowing our effect estimates to vary between age strata, for a reliable estimate of the effect of RASIs in the older stratum of the Covid-19 population. Finally, we have compared mortality between treatment groups that were rigorously matched with respect to 26 medical history variables, by using advanced propensity score methods.

## MATERIALS AND METHODS

### Ethics

Necessary approval was obtained from the Bergamo Ethics Committee (no. 37/2020) with operating centre at the Papa Giovanni XXIII Hospital of Bergamo. In conformity with local protocol, informed consent was obtained from the patients.

### Data

We included in our study all patients older than 18 years with history of hypertension with positive rhino-pharyngeal swab for SARS-CoV-2 infection, hospitalized at Papa Giovanni XXIII Hospital (a tertiary hospital of 1080 beds located in Bergamo, the initial epicentre of Italian Covid-19 storm) with a diagnosis of Covid-19 based on the updated WHO interim guidance document [11], between 23 February and 7 April 2020. Conversely, milder degrees of the disease state, not requiring hospitalization, were not included in the present analysis. Patient's follow-up ended on 5 May 2020. Information about the history and physical examination of these patients was derived via chart review by medical officers. Chart reviews were performed by two independent doctors. This type of adjudication of data provided quality checks. In case of disagreement regarding a specific item, a third doctor had to review that item to provide internal consistency. Variables collected through standardized recording forms included age, sex, comorbidities, dates of symptoms’ onset and hospital admission. Hypertension was defined as having a history of DBP equal or greater than 90 mmHg and/or a SBP equal to or greater than 140 mmHg and/or a history of antihypertensive medication use. Laboratory confirmation of SARS-CoV-2 infection via SARS-CoV-2 genome detection from nasal swab and respiratory samples was obtained through two different molecular methods (GeneFinder COVID-19-Elitech Group, Allplex 2019-nCoV Assay-Seegene Inc) following instructions. After purification of viral RNA from clinical samples, presence of RdRp, E and N viral genes was detected by using real-time PCR (RT-PCR) according to WHO protocol.

### Outcome

Primary endpoint was mortality from all-causes, either occurring in-hospital or within 1 month after discharge.

### Variables

Data were subjected to quality checks, validated for internal consistency and then anonymized prior to transfer.

### Statistical analysis

Descriptive statistics were used to summarize characteristics of hypertensive Covid-19 patients. The chi-square test (or Fisher's exact test, when appropriated) was used to compare categorical variables of the two groups, the Wilcoxon-Mann-Whitney test was used to compare continuous variables. No sample size calculations were performed. Age was dichotomized via median split as less than 68 or at least 68 years, with no attempt to optimize the divide. The cutoff of 68 years was defined on the basis of the median age of the whole Covid-19 population. As Covid-19 cases with hypertension did not significantly differ in terms of many comorbidities and characteristics, according to the 68 cut-off (which is the median age of the whole population) or the 72 cut-off (which is the median age of the hypertensive), as reported in Supplementary Table 1, we decided to be more conservative maintaining the original cut-off of 68 years, in order to avoid the increase in false positives due to the to shift of the threshold chosen. Importantly, we have not optimized the age threshold in our analysis of hypertensive not to inflate Type-1 error. The symbol 68+ will hereafter stand for at least 68 years. Missing values were imputed via R package MICE. No signs of systematic missingness were detected. Obesity and smoking were excluded from the main analysis due to a percentage of missing values in excess of 5%. Results from a subsidiary analysis restricted to the set of patients with complete information about these two variables, and performed by including these into the models, did not yield appreciably different results from the main analysis.

Overall survival on the total sample of hypertensive patients according to age and previous exposure to RASIs (four groups of patients: age <68 and no-RASIs use, age <68 and RASIs use, age 68+ and no-RASIs use, age 68+ and RASIs use) was estimated by the Kaplan--Meier method. The corresponding group-specific curves were compared by the log-rank test. Subsequently, with the aim to reduce confounding and to create two comparable groups on the basis of prehospitalization characteristics, we constructed a propensity score model for antecedent use of RASIs based on the variables reported in Table 1 (except for the survival indicator) [12,13]. The model parameter estimates were used to calculate a value of this score for each sample patient. Two subgroups of patients, RASIs-exposed versus RASIs-free, were created by matching with respect to the score, and compared in terms of mortality by a logistic regression of the survival outcome on the RASIs exposure indicator, also allowing this indicator and the binary (<68 versus 68+) age indicator to interact in their effects on the outcome. Effect of continuous age was modelled non-parametrically via splines.

**TABLE 1:** Characteristics of the 688 hypertensive Covid-19 patients stratifying by RASIs use versus no-RASIs use

		Total	No-RASIs use	RASIs use	
	*N* non-missing	*N* = 688	*N* = 229	*N* = 459	*P*
Male sex	688	489 (71.1%)	154 (67.2%)	335 (73.0%)	0.12
Age, median (IQR)	688	72.0 (63.0–79.0)	73.0 (64.0–79.0)	72.0 (63.0–79.0)	0.35
BMI, median (IQR)	503	27.3 (24.6–30.7)	26.8 (24.3–30.0)	27.4 (24.8–31.1)	0.20
Obesity (BMI > 30)	512	149 (29.1%)	43 (25.3%)	106 (31.0%)	0.18
Smoking history					
Current smoker	606	20 (3.3%)	10 (5.2%)	10 (2.4%)	0.096
Former smoker		144 (23.8%)	51 (26.4%)	93 (22.5%)	
Never smoker		442 (72.9%)	132 (68.4%)	310 (75.1%)	
Comorbidities					
Diabetes	686	193 (28.1%)	54 (23.6%)	139 (30.4%)	0.060
CKF	685	84 (12.3%)	33 (14.4%)	51 (11.2%)	0.22
COPD	685	62 (9.1%)	28 (12.2%)	34 (7.5%)	0.040
Long-term oxygen therapy	685	18 (2.6%)	5 (2.2%)	13 (2.9%)	0.61
Active solid neoplasm	684	29 (4.2%)	11 (4.8%)	18 (4.0%)	0.60
Active haematologic malignancy	684	24 (3.5%)	12 (5.2%)	12 (2.6%)	0.12
Cerebrovascular disease	684	54 (7.9%)	23 (10.1%)	31 (6.8%)	0.13
Previous myocardial infarction	683	101 (14.8%)	33 (14.5%)	68 (14.9%)	0.87
Chronic heart failure	686	47 (6.9%)	19 (8.3%)	28 (6.1%)	0.29
Angina/previous revascularization	671	109 (16.2%)	37 (17.1%)	72 (15.9%)	0.70
Atrial fibrillation	674	93 (13.8%)	33 (15.1%)	60 (13.2%)	0.49
Vasculopathy	686	91 (13.3%)	30 (13.1%)	61 (13.3%)	0.93
Rheumatic disease	685	38 (5.5%)	12 (5.2%)	26 (5.7%)	0.80
Immunosuppression	684	39 (5.7%)	15 (6.6%)	24 (5.3%)	0.48
Home therapies					
MRAs	652	44 (6.7%)	22 (10.8%)	22 (4.9%)	0.006
Loop diuretics	652	135 (20.7%)	56 (27.5%)	79 (17.6%)	0.004
Other diuretics	651	132 (20.3%)	14 (6.9%)	118 (26.4%)	<0.001
Beta-blockers	650	266 (40.9%)	93 (46.0%)	173 (38.6%)	0.075
Calcium channel blockers	688	332 (48.3%)	134 (58.5%)	198 (43.1%)	<0.001
Statins	651	242 (37.2%)	63 (31.0%)	179 (40.0%)	0.029
Steroids	681	35 (5.1%)	13 (5.8%)	22 (4.8%)	0.61
Oral antidiabetics	682	141 (20.7%)	38 (16.8%)	103 (22.6%)	0.080
Insulin	682	51 (7.5%)	17 (7.5%)	34 (7.5%)	0.98
OAT/DOACs	683	112 (16.4%)	42 (18.6%)	70 (15.3%)	0.28
Antiplatelets	683	254 (37.2%)	87 (38.5%)	167 (36.5%)	0.62
Proton pump inhibitors	681	263 (38.6%)	88 (38.9%)	175 (38.5%)	0.90
Symptoms on admission					
Fever	679	581 (85.6%)	189 (83.6%)	392 (86.5%)	0.31
Cough	678	264 (38.9%)	94 (41.8%)	170 (37.5%)	0.29
Anorexia	678	48 (7.1%)	15 (6.7%)	33 (7.3%)	0.77
Asthenia	678	187 (27.6%)	66 (29.3%)	121 (26.7%)	0.47
Myalgia	678	39 (5.8%)	15 (6.7%)	24 (5.3%)	0.47
Dyspnoea	678	431 (63.6%)	141 (62.7%)	290 (64.0%)	0.73
Sore throat	677	11 (1.6%)	4 (1.8%)	7 (1.5%)	0.76
Dizziness	678	26 (3.8%)	7 (3.1%)	19 (4.2%)	0.49
Abdominal pain	678	16 (2.4%)	6 (2.7%)	10 (2.2%)	0.71
Diarrhoea	678	62 (9.1%)	19 (8.4%)	43 (9.5%)	0.66
Nausea	677	36 (5.3%)	10 (4.4%)	26 (5.8%)	0.48
Vomiting	678	34 (5.0%)	10 (4.4%)	24 (5.3%)	0.63
Chest pain	678	27 (4.0%)	9 (4.0%)	18 (4.0%)	0.99
Hypo/anosmia	668	8 (1.2%)	0 (0.0%)	8 (1.8%)	0.059
Hypo/agenusia	669	12 (1.8%)	0 (0.0%)	12 (2.6%)	0.012
Vital signs at entry					
Heart beat frequency (bpm)	604	83.0 (73.0–93.0)	81.0 (73.0–91.0)	84.0 (73.0–94.0)	0.22
Systolic blood pressure (mmHg)	593	127.0 (113.0–142.0)	125.0 (110.0–140.0)	129.0 (115.0–145.0)	0.032
Outcome					
Death	688	240 (34.9%)	85 (37.1%)	155 (33.8%)	0.39

Symbol *N* stands for group numerosity. Symbol *P* stands for *P* value for the difference between RASIs-use and no-RASIs-use populations with respect to a specific characteristic. For each yes-no characteristic (e.g. male sex), the table reports number and percentage of ‘yes’ patients within a particular stratum. CKF, chronic kidney failure, defined as glomerular filtration rate <60 ml/min per m^2^; COPD, chronic obstructive pulmonary disease; MRAs, mineralocorticoid receptor antagonist; OAT/DOACs, oral anticoagulant therapy/direct oral anticoagulants.

A significantly different value of this parameter from zero would represent evidence of the two effects interacting on a multiplicative scale. Because interaction is more relevant to public health if expressed on an additive scale [14], in our study, we present evidence of RASIs × age interaction also in a relative excess risk (RER) form [13–15] after appropriate dichotomization of the continuous age variable (≤68 versus 68+). A positive RER is obtained wherein there is a “target” age stratum wherein a real-world intervention in favour of RASIs is likely to have greater impact than in remaining population.

Finally, we performed an analysis on the subgroup of hypertensive patients older than 68 years considering two RASIs drug subclasses, ACEIs and ARBs, and assessed their effects by taking no-antecedent-use of RASIs as reference. Pairwise comparisons between these groups in terms of mortality from Covid-19 were again performed by using propensity score matching methods [12–16] to make these groups comparable with respect to potential prehospitalisation confounders, specifically, chronic use of medications and pre-existing comorbidities. Effects of interest were estimated via logistic regression of the binary survival outcome on the exposure variable of interest.

An α level of 0.05 was used for all hypothesis tests.

Analyses were conducted with R software (URL https://www.R-project.org/).

## RESULTS

From our initial sample of 1352 patients admitted for Covid19 (characteristics reported in Table S2), we considered 688 hypertensive patients. Follow-up time had a median of 34 days and an interquartile range (IQR) of 19–41. There were overall 240 (35%) deaths. Table 1 summarizes demographic, home therapy and comorbidity data of our hypertensive patients, and compares the 459 patients on RASIs at admission with the remaining 229. A total of 489 patients (71.1%) were men; median age was 72 years (IQR: 63–79). Hypertensive RASIs users were similar to non-users in terms of comorbidities, with the exception of a higher frequency of patients with COPD in the group of non-users (12.2 versus 7.5%, *P* = 0.040). Moreover, hypertensive no-RASIs users were also more frequently treated with mineralocorticoid-receptor antagonists, diuretics, betablockers and calcium channel blockers, while hypertensive RASIs users were more frequently treated with statins. Supplementary table S3 displays the characteristics of RASIs users and no-RASIs users according to the age cut-off of 68 years.

**FIGURE 1:**
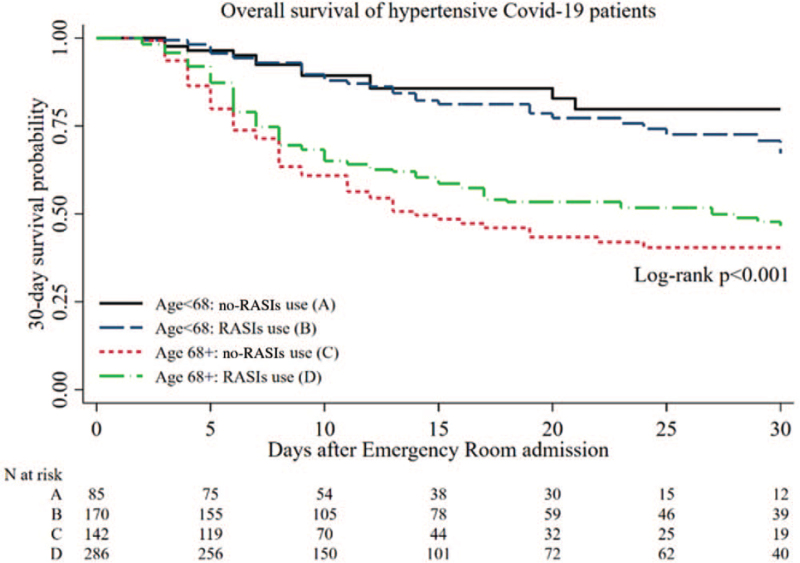
Kaplan--Meier 30-day survival curves of the 688 hypertensive Covid-19 patients according to age strata and RASIs use.

Figure 1 shows the Kaplan–Meier 30-day survival curves on the total sample of 688 hypertensive patients according to age and previous exposure to RASIs. There was a significant difference (log-rank *P* < 0.001) in survival among the four groups of patients (best survival for younger individuals age <68, independently from RASIs use, while among age 68+ individuals better survival for RASIs user than for no-RASIs users) in spite of the fact that Kaplan–Meier estimator might underestimate RASIs effect because it does not fully adjust for confounding variables. In particular, the different effect of RASIs in the two age strata suggests a possible interaction, which was subsequently tested in the propensity-score matching analysis.

In the remaining part of our analysis, we confirm and strengthen this finding using a propensity score matching analysis and assess the above association at the level of individual RASIs drug subclasses: ACEIs and ARBs.

On the basis of the variables in Table 1 (except for the survival indicator), we constructed a propensity score model for antecedent use of RASIs. Among the variables considered in the propensity score model, there were COPD and diuretic use, to account for the higher incidence in the non-RAS blocker group. Effect of continuous age was modelled non-parametrically via splines. The model parameter estimates were used to calculate a value of this score for each sample patient. Two subgroups of patients, RASIs-exposed versus RASIs-free, were created by matching with respect to the score, and compared in terms of mortality by a logistic regression of the survival outcome on the RASIs exposure indicator, also allowing this indicator and the binary (*<*68 versus 68+) age indicator to interact in their effects on the outcome. There was significant evidence of interaction (*P* = 0.001). Moreover, from this model, we found that older Covid-19 patients with hypertension gain more (in terms of survival) from prior exposure to RASIs than their younger counterparts, corresponding to a positive RER (0.19).

In a sensitivity analysis considering also normotensive individuals (*N* = 1352), results were consistent; 71.6% of the sample were men; 50.9% had arterial hypertension; 19.4% (*N* = 262) were treated with ACEIs and 17.2% (*N* = 232) with ARBs. During follow-up, there were 353 (26.1%) deaths. Age, dichotomized as 68 or less or more than 68 years was found to modify the effect of RASIs (*P* < 0.001), and RASIs were found to be protective [odds ratio (OR) 0.71, 95% confidence interval (95% CI) 0.51–0.98, *P* = 0.03] among patients aged more than 68 years. Among these older patients, ARBs use was associated with lower mortality (OR 0.52, 95% CI 0.32–0.83, *P* = 0.006), with ACEIs exhibiting a nonsignificant trend towards a similar effect (OR 0.68, 95% CI 0.43–1.06, *P* = 0.09).

**FIGURE 2:**
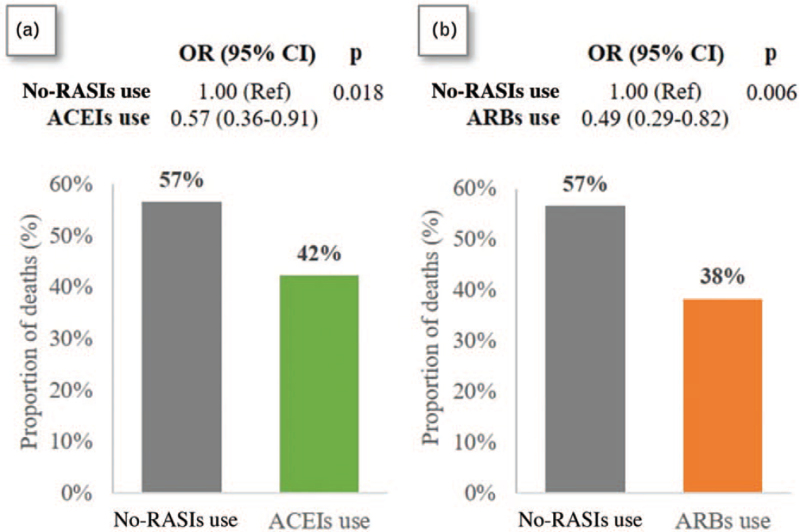
Propensity-matched analysis on the effect of ACEIs (box a) or ARBs (box b) use vs no-RASIs use on mortality among hypertensive Covid-19 patients over 68 years of age.

Figure 2 reports the two propensity-matched comparisons between the 68+ hypertensive Covid-19 patients: box A and B report the matched comparisons between no-RASIs use versus ACEIs use (and no ARBs) and between no-RASIs use versus ARBs use (and no ACEIs), respectively, in terms of crude mortality estimates (bar graphs) and of adjusted ORs (and 95% CIs) obtained by the corresponding logistic regression models. In each comparison, the two exposures groups were matched for the relevant propensity score. Each reported OR estimate is based on the reduced samples produced by the matching (144 per exposure group in the assessment of ACEIs versus no-RASIs use and 120 per exposure group in the assessment of ARBs versus no-RASIs use). Both effects appear to be significantly different from zero. In particular, ACEIs effect appears lower in magnitude (higher OR) than that of ARBs, but not significantly so. These results may be summarized as follows: antecedent use of ACEIs, when compared with no-RASIs use within the stratum of 68+ hypertensive Covid-19 patients, was significantly associated with a lower mortality (*P* = 0.018, OR = 0.57, 95% CI 0.36–0.91), after adjusting for medical history via propensity matching. Similarly, among the subgroup of 68+ hypertensive Covid-19 patients, previous use of ARBs was significantly associated with a lower mortality (*P* = 0.006, OR = 0.49, 95% CI 0.29–0.82) with respect to no-RASIs use.

**FIGURE 3:**
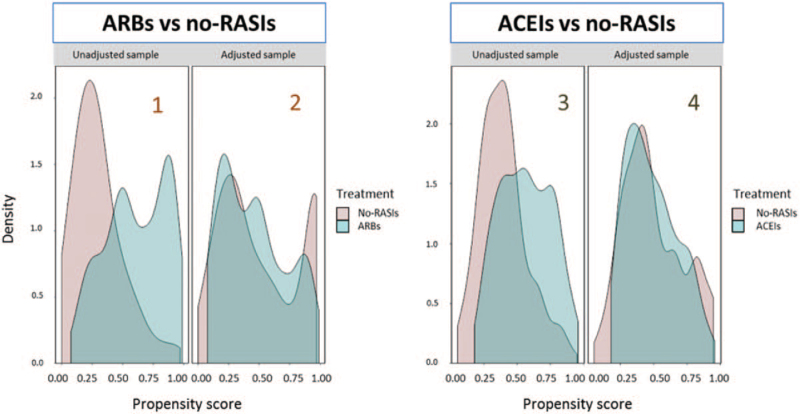
Visual assessment of the degree of balancing achieved by the matching procedure in our analyses of the effects of ARBs (two plots in the left half of the figure) and of ACEIs (two plots in the right half) on mortality in the population of 68+ Covid-19 patients with arterial hypertension. Moving from left to right, plots 1 and 2 show the estimated densities of the propensity score for ARBs use within the groups of ARBs users (blue) and no-RASIs users (pink), before (plot 1) and after (plot 2) the matching. Plots 3 and 4 show the corresponding data for ACEIs.

For purposes of illustration, effectiveness of the matching in our analysis of ARBs effect can be visually appreciated in Fig. 3. Although the unmatched exposure groups were not comparable in term of medical history and comorbidities, the matching has played a crucial role in creating conditions for a credible estimate of ARBs effect on mortality in the population of 68+ Covid-19 patients with arterial hypertension. Completely analogous remarks can be made in relation with our assessment of the effect of pre-hospitalization exposure to ACEIs on mortality in the population of 68+ Covid-19 patients with arterial hypertension. Additional details regarding comparability between two matched ARBs and no-RASIs groups (120 patients each) in terms of clinical observations, biochemical parameters collected upon hospital admission, age, comorbidities and chronic therapies are provided in the Supplementary appendix (Tables S4, S5, S6 and S7).

Among 355 RASIs users at hospital admission for which we have data during hospitalization, 25.4% of ARBS users and 30% of ACEIs users continued their therapy with no significant difference in mortality as compared to those who discontinued such therapy.

## DISCUSSION

Our study, through the analysis of data derived from a large cohort in the Northern Italy area, provides evidence that antecedent-use of RASIs, either ACEIs or ARBs, in a population of over-68 hypertensive Covid-19 patients is associated with a lower all-cause mortality, whether in-hospital or shortly after discharge, compared with no-RASIs use. Importantly, the association between antecedent use of RASIs and better Covid-19 outcome is observed provided we focus on the higher risk stratum of elderly hypertensive patients. Our data do not provide evidence of any RASIs effect on outcome in similar, but younger, patients.

The protective effect found in the elderly may possibly be explained by the ability of RASIs to avert COVID-induced cardiovascular complications more frequent in this age group and in comorbid patients [17–20]. In fact, RASIs antagonize the deleterious effects of Ang II. Liu *et al*. [21] report serum Ang II plasma levels in a sample of 12 Covid-19 infected patients as being markedly elevated and linearly associated with viral load and lung injury. These findings support the hypothesis that elevated levels of Ang II may foster acute respiratory distress syndrome (ARDS) in Covid-19 patients, which would explain the protective role of RASIs found in older Covid-19 patients. In addition, in-vitro cells treatment with Ang II was found to enhance ACE2 ubiquitination also mediated by AT1R, ultimately stimulating ACE2 lysosomal degradation [22]. This might prevent interaction of SARS-CoV-2 with ACE2 catalytic site. Noteworthily ARBs, through AT1R antagonism, have been suggested as drugs able to prevent virus/ACE2 interaction, such pathway representing a putative mechanism by which ARBs, more than ACEIs, might prevent SARS-CoV-2 cells entry [23]. Another hypothesis, maybe speculative, suggests that the beneficial effect of previous RASIs use is related to higher ACE2 expression with aging [24]. Thus, the older the patient the higher might ACE2 expression be and, concordantly, the greater might the RASIs’ beneficial effect in Covid-19 be. Finally, RASIs have antithrombotic properties that could further ameliorate the clinical course of Covid-19, possibly by reducing the thromboembolic complications associated with this disease [25–27].

Unlike previous works, the present study avoids a serious methodological misstep by incorporating effect modification due to age, in such a way to try to avoid effect estimate dilution. Such a misstep might be a reason why results from a number of previous studies point in the same direction as ours without achieving nominal statistical significance, a notable example being the study by Fosbol *et al*. [4]. The important and large, population-based, study by Mancia *et al*. [5] finds no evidence that ACEIs or ARBs affect risk of Covid-19. This result does not exclude ours. In fact, little detail is given in that paper about the “multivariable adjustment” procedure used to calculate the effects of interest. In addition, the study relies on administrative data from regional databases with possibly incomplete information on comorbidities and drug use. More importantly, the outcome in study by Mancia *et al*. [5] is diagnosis of severe Covid-19, rather than mortality. Of the four possible ‘coexisting conditions’ that characterize individuals in their analysis (‘respiratory disease’, ‘cardiovascular disease’, ‘kidney disease’ and ‘cancer’), only the first turns out to be characterized by a significant effect according to that study. In one of these studies [9], only a few variables are considered for adjustment in the final model (age, BMI, renal function on admission, and use of RASIs). In addition, these authors include normotensive patients in their analysis, which raises problems of comparability, and they avoid adjusting for age on a continuous scale within each broader age stratum. Finally, low statistical power appears to prevent these authors from separately considering the effects of ACEIs and ARBs. Another study [10] finds significant evidence of a protective effect of antecedent use of ACEIs in hospitalized Covid-19 patients, and nonsignificant evidence of a role for antecedent use of ARBs, but this conclusion could be a consequence of the smaller size of their ARB-using sample. Moreover, they do not confine assessment of RASIs effect to the population of hypertensive individuals, which raises again the comparability issue [10].

Our study was possible because RASIs drug allocation in the general population did not follow a fixed and uniform protocol based on the individual's characteristics. In other words, primary care RASIs prescription was relatively liberal. This is reflected by Fig. 3, which shows that for each RASIs-exposed patient, we could find a patient with similar propensity score who had not used RASIs, for balanced comparison of the two treatment groups. An open question is whether the protective effects of RASIs are to be ascribed to their use before or after the individual becomes infected, or perhaps to both timings. Resolving this argument is beyond the scope of the present work. Under an observational regimen, post-hospitalization administration of RASIs will be associated to prior exposure to the same drug and it will depend on decisions involving unrecorded information. Some authors concentrate on the effect of prior use of RASIs on patient admission parameters that appear to predict a severe outcome, under the assumption that those are causal parameters. Inference about the effect of post-admission therapeutic decisions should, ideally, be made via randomized studies, although there could be an ethical objection to randomizing assignment of a drug when an observational study has shown that the drug is likely to be beneficial. Nevertheless, a RCT to assess the effect of continuing/discontinuing RASIs in patients hospitalized for Covid-19 has been performed, the BRACE CORONA trial [28]. This trial assessed the effect of discontinuing RASIs on Covid-19 outcome. One problem with RCTs in a climate of health care urgency is that they require time. In order to circumvent this problem, some studies fix a short follow-up horizon, such as 30 days in BRACE CORONA. Such a short time span, however, may work only with a cohort of individuals at a very high risk of severe outcome, which was not the case in BRACE CORONA. In fact, BRACE CORONA, with a mortality rate of only 2.7%, recorded only nine deaths per study arm. In spite of the low number of events, results from BRACE CORONA show a tendency towards a survival advantage of ACEIs/ARBs use. In the light of results from our study, it is not unreasonable to conjecture that had BRACE CORONA restricted admission to an old age stratum, or more in general to high-risk patients, their results would have been in accord with ours. By providing evidence of a beneficial effect of both ARBs and ACEIs, our study may be taken as suggesting that future RCTs should shine a light on both these drug classes.

### Strength and limitations

Our study was based on a single big hospital. Although collaborative data from multi-hospital observational data analysis would have allowed us to consider a broader population with Covid-19, this may also be viewed as an element of strength of the study, insofar as homogeneity of target population reduces potential biases. Two characteristics of our cohort, high percentage of elderly hypertensive individuals and peak Covid-19 lethality, enhanced our power to detect the effects of interest. We have used propensity-score matching methods to create exposure comparison groups that are comparable with respect to observed potential confounders. The separate analyses according to ACEIs and ARBs comprise a small sample of patients. However, due to the rigorous statistical approach applied, we were able to obtain matched subgroups with and without ACEIs or ARBs, providing some novel data. Despite the rather large number of medical history variables involved in the construction of our propensity scores, there may be additional unmeasured confounders that have not been taken into account, and consequently affect our results. Post-discharge follow-up was limited in time. Finally, our study was not designed to systematically explore the effects on mortality of continuing or discontinuing RASIs during hospitalization. However, on the basis of data available in our patients, no difference in mortality was observed between those who continued and those who discontinued RASIs during hospital admission. In addition, short-term temporary withdrawal of RASIs during Covid-19 hospitalization cannot be considered equivalent to the absence of previous drug exposure, in view of their long-lasting structural and functional cardiovascular and renal effects.

In conclusion, our study provides direct support to International, European and American expert consensus statements [29,30] recommending not to withdraw RASIs in patients with Covid-19 and associated cardiovascular conditions. Our data might support further research to assess whether initiation of RASIs in elderly hypertensive patients could protect against Covid-19 adverse outcome.

## ACKNOWLEDGEMENTS

Funding for this study was provided from Research Foundation of the Bergamo Hospital;

Clinical and Pharmacological Research Project for a Covid-19 Treatment ‘3x1’.

M.G., E.D. did data collection, literature search, writing and design of present document.

C.B., L.B. did statistical design of study, data analysis, literature search, writing and design of present document.

A.G. did data analysis supervision, literature search, writing and study design.

A.G., G.P., M.S., S.F., F.L.L., G.G., F.D.M., R.T. did the supervision, literature search, writing and study design; G.B., A.G., A.B., M.A., F.R., L.N., B.M., G.M. did the data collection.

### Conflicts of interest

M.G. received consulting fees from Novartis, Boehringer and Menarini

R.T. received lecture fees from AstraZeneca, Boehringer Ingelheim, Eli Lilly, Novo Nordisk and Sanofi, and has participated in advisory panels for AstraZeneca, Boehringer Ingelheim, Eli Lilly, Novo Nordisk and Sanofi.

S.F. is in the speaker's bureau and advisory board for Gilead, Abbvie, MSD, Novartis, Astellas, Intercept, Kedrion, Bayer.

M.S. received consulting fees from Novartis, Merck, Bayer, Vifor Pharma, Abbott, Boehringer Ingelheim, Astra-Zeneca, Servier.

G.P. received honoraria for lectures by Omron HealthCare, Servier, Fidia.

The remaining authors have no conflicts of interest.

## Supplementary Material

Supplemental Digital Content
