# Supplementary material for: Antecedent use of renin-angiotensin system inhibitors is associated with reduced mortality in elderly hypertensive Covid-19 patients
Source: J Hypertens. 2021 Dec 9;40(4):666–74. doi: 10.1097/HJH.0000000000003059 (PMC9904437; doi:10.1097/HJH.0000000000003059)
Supplement: Supplemental Digital Content [file jhype-40-666-s001.doc]

**SUPPLEMENTARY MATERIAL**

**Supplementary Appendix** to “Antecedent Use of Renin-Angiotensin System Inhibitors Is Associated with Reduced Mortality of Older Hypertensive Covid-19 Patients”

**Table S1**. Comparison of characteristics of hypertensive Covid-19 patients according to different age cut-off.

|  | **N non missing** | **< 68y** | **≥ 68y** | **p** | **< 72y** | **≥ 72y** | **p** |
| --- | --- | --- | --- | --- | --- | --- | --- |
|  |  | **N=256** | **N=432** |  | **N=347** | **N=341** |  |
| **RASI exposure** |  | 171 (66.8%) | 288 (66.7%) | 0.97 | 235 (67.7%) | 224 (65.7%) | 0.57 |
| Male gender | 688 | 196 (76.6%) | 293 (67.8%) | 0.015 | 262 (75.5%) | 227 (66.6%) | 0.010 |
| Age,  *median (IQR)* | 688 | 60.5  (55.0-64.0) | 77.0  (73.0-82.0) | <0.001 | 63.0  (58.0-69.0) | 79.0  (75.0-83.0) | <0.001 |
| BMI, *median (IQR)* | 503 | 27.8  (25.1-31.7) | 26.7  (24.2-30.1) | 0.002 | 27.8  (25.0-31.5) | 26.1  (24.2-29.4) | <0.001 |
| Obesity (BMI > 30) | 512 | 69 (34.2%) | 80 (25.8%) | 0.042 | 97 (36.1%) | 52 (21.4%) | <0.001 |
| **Smoking history** |  |  |  |  |  |  |  |
| Current smoker | 606 | 9 (3.9%) | 11 (2.9%) | 0.50 | 13 (4.2%) | 7 (2.3%) | 0.41 |
| Former smoker |  | 49 (21.5%) | 95 (25.1%) |  | 71 (23.2%) | 73 (24.3%) |  |
| Never smoker |  | 170 (74.6%) | 272 (72.0%) |  | 222 (72.5%) | 220 (73.3%) |  |
| **Comorbidities** |  |  |  |  |  |  |  |
| Diabetes | 686 | 59 (23.2%) | 134 (31.0%) | 0.028 | 87 (25.2%) | 106 (31.1%) | 0.087 |
| CKF | 685 | 18 (7.1%) | 66 (15.3%) | 0.002 | 26 (7.5%) | 58 (17.1%) | <0.001 |
| COPD | 685 | 14 (5.5%) | 48 (11.1%) | 0.013 | 24 (7.0%) | 38 (11.2%) | 0.054 |
| Long-term oxygen therapy | 685 | 6 (2.4%) | 12 (2.8%) | 0.74 | 7 (2.0%) | 11 (3.2%) | 0.32 |
| Active solid neoplasm | 684 | 11 (4.3%) | 18 (4.2%) | 0.93 | 13 (3.8%) | 16 (4.7%) | 0.54 |
| Active hematologic malignancy | 684 | 9 (3.5%) | 15 (3.5%) | 1.00 | 12 (3.5%) | 12 (3.5%) | 1.00 |
| Cerebrovascular disease | 684 | 15 (5.9%) | 39 (9.1%) | 0.14 | 27 (7.8%) | 27 (8.0%) | 0.95 |
| Previous Myocardial Infarction | 683 | 25 (9.8%) | 76 (17.7%) | 0.005 | 42 (12.2%) | 59 (17.4%) | 0.056 |
| Chronic heart failure | 686 | 9 (3.5%) | 38 (8.8%) | 0.008 | 12 (3.5%) | 35 (10.3%) | <0.001 |
| Angina/previous revascularization | 671 | 26 (10.3%) | 83 (19.9%) | 0.001 | 46 (13.5%) | 63 (19.1%) | 0.049 |
| Atrial fibrillation | 674 | 14 (5.5%) | 79 (18.8%) | <0.001 | 23 (6.7%) | 70 (21.1%) | <0.001 |
| Vasculopathy | 686 | 21 (8.2%) | 70 (16.2%) | 0.003 | 30 (8.7%) | 61 (17.9%) | <0.001 |
| Rheumatic pathology | 685 | 8 (3.1%) | 30 (7.0%) | 0.035 | 14 (4.1%) | 24 (7.1%) | 0.086 |
| Immunosuppression | 684 | 20 (7.9%) | 19 (4.4%) | 0.057 | 24 (7.0%) | 15 (4.4%) | 0.15 |
| **Home therapies** |  |  |  |  |  |  |  |
| MRAs | 652 | 10 (4.1%) | 34 (8.3%) | 0.037 | 19 (5.8%) | 25 (7.8%) | 0.31 |
| Loop diuretics | 652 | 28 (11.5%) | 107 (26.2%) | <0.001 | 47 (14.2%) | 88 (27.3%) | <0.001 |
| Other diuretics | 651 | 46 (18.9%) | 86 (21.1%) | 0.51 | 65 (19.8%) | 67 (20.8%) | 0.74 |
| Beta-blockers | 650 | 79 (32.5%) | 187 (45.9%) | <0.001 | 113 (34.3%) | 153 (47.7%) | <0.001 |
| Calcium channel blockers | 688 | 119 (46.5%) | 213 (49.3%) | 0.47 | 157 (45.2%) | 175 (51.3%) | 0.11 |
| Statins | 651 | 64 (26.2%) | 178 (43.7%) | <0.001 | 105 (31.8%) | 137 (42.7%) | 0.004 |
| Steroids | 681 | 10 (4.0%) | 25 (5.8%) | 0.28 | 17 (5.0%) | 18 (5.3%) | 0.83 |
| Oral antidiabetics | 682 | 48 (19.0%) | 93 (21.7%) | 0.40 | 68 (19.8%) | 73 (21.6%) | 0.56 |
| Insulin | 682 | 14 (5.5%) | 37 (8.6%) | 0.14 | 19 (5.5%) | 32 (9.5%) | 0.050 |
| OAT/DOACs | 683 | 19 (7.5%) | 93 (21.6%) | <0.001 | 33 (9.6%) | 79 (23.3%) | <0.001 |
| Antiplatelets | 683 | 52 (20.6%) | 202 (47.0%) | <0.001 | 87 (25.3%) | 167 (49.3%) | <0.001 |
| Proton Pump inhibitors | 681 | 65 (25.9%) | 198 (46.0%) | <0.001 | 100 (29.2%) | 163 (48.1%) | <0.001 |
| **Symptoms on admission** |  |  |  |  |  |  |  |
| Fever | 679 | 223 (88.5%) | 358 (83.8%) | 0.096 | 299 (87.2%) | 282 (83.9%) | 0.23 |
| Cough | 678 | 124 (49.2%) | 140 (32.9%) | <0.001 | 153 (44.6%) | 111 (33.1%) | 0.002 |
| Anorexia | 678 | 16 (6.3%) | 32 (7.5%) | 0.57 | 22 (6.4%) | 26 (7.8%) | 0.49 |
| Asthenia | 678 | 58 (23.0%) | 129 (30.3%) | 0.041 | 87 (25.4%) | 100 (29.9%) | 0.19 |
| Myalgia | 678 | 19 (7.5%) | 20 (4.7%) | 0.12 | 25 (7.3%) | 14 (4.2%) | 0.082 |
| Dyspnoea | 678 | 171 (67.9%) | 260 (61.0%) | 0.074 | 228 (66.5%) | 203 (60.6%) | 0.11 |
| Sore throat | 677 | 6 (2.4%) | 5 (1.2%) | 0.35 | 8 (2.3%) | 3 (0.9%) | 0.22 |
| Dizziness | 678 | 7 (2.8%) | 19 (4.5%) | 0.27 | 8 (2.3%) | 18 (5.4%) | 0.039 |
| Abdominal pain | 678 | 7 (2.8%) | 9 (2.1%) | 0.58 | 11 (3.2%) | 5 (1.5%) | 0.14 |
| Diarrhoea | 678 | 21 (8.3%) | 41 (9.6%) | 0.57 | 31 (9.0%) | 31 (9.3%) | 0.92 |
| Nausea | 677 | 13 (5.2%) | 23 (5.4%) | 0.90 | 19 (5.6%) | 17 (5.1%) | 0.78 |
| Vomiting | 678 | 10 (4.0%) | 24 (5.6%) | 0.34 | 16 (4.7%) | 18 (5.4%) | 0.67 |
| Chest pain | 678 | 10 (4.0%) | 17 (4.0%) | 0.99 | 12 (3.5%) | 15 (4.5%) | 0.51 |
| Hypo/anosmia | 668 | 6 (2.4%) | 2 (0.5%) | 0.058 | 6 (1.8%) | 2 (0.6%) | 0.29 |
| Hypo/agenusia | 669 | 7 (2.8%) | 5 (1.2%) | 0.23 | 8 (2.3%) | 4 (1.2%) | 0.38 |
| **Vital signs at entry** |  |  |  |  |  |  |  |
| Heart Beat Frequency (bpm)  *median (IQR)* | 604 | 87.0  (77.0-95.0) | 80.0  (71.0-90.0) | <0.001 | 86.0  (76.0-95.0) | 80.0  (70.0-89.0) | <0.001 |
| Systolic blood pressure (mmHg)  *median (IQR)* | 593 | 126.0  (113.0-140.0) | 127.0  (113.0-144.0) | 0.65 | 126.0  (112.0-140.0) | 128.0  (115.0-145.0) | 0.14 |
| **Outcome** |  |  |  |  |  |  |  |
| Death | 688 | 46 (18.0%) | 194 (44.9%) | <0.001 | 79 (22.8%) | 161 (47.2%) | <0.001 |

BMI: Body mass index

CKF: Chronic kidney failure, defined as glomerular filtration rate <60 ml/min/m^2^.

COPD: chronic obstructive pulmonary disease

MRAs: mineralocorticoid receptor antagonists

OAT/DOACs: oral anticoagulant therapy/direct oral anticoagulants

**Table S2. Characteristics of the total 1,352 Covid-19 patients stratifying by RASIs-use vs no RASIs-use. Symbol N stands for group numerosity. Symbol p stands for p-value for the difference between RASIs-use and no-RASIs-use populations with respect to a specific characteristic. For each yes-no characteristic (eg., male gender) the table reports number and percentage of "yes" patients within a particular stratum.**

|  | **N non missing** | **Total** | **No RASIs use** | **RASIs use** | **P** |
| --- | --- | --- | --- | --- | --- |
|  |  | **N=1,352** | **N=866** | **N=486** |  |
| Male gender | 1,352 | 968 (71.6%) | 609 (70.3%) | 359 (73.9%) | 0.17 |
| Age, *median (IQR)* | 1,352 | 68.0 (57.0-76.0) | 64.0 (54.0-74.0) | 72.0 (63.0-79.0) | <0.001 |
| Body mass index (BMI), *median (IQR)* | 991 | 26.5 (24.4-29.6) | 26.2 (24.2-29.3) | 27.4 (24.8-31.0) | <0.001 |
| Obesity (BMI > 30) | 1,005 | 242 (24.1%) | 132 (20.5%) | 110 (30.5%) | <0.001 |
| **Smoking history** |  |  |  |  |  |
| Current smoker | 1,198 | 49 (4.1%) | 38 (5.0%) | 11 (2.5%) | 0.071 |
| Former smoker |  | 249 (20.8%) | 150 (19.8%) | 99 (22.5%) |  |
| Never smoker |  | 900 (75.1%) | 570 (75.2%) | 330 (75.0%) |  |
| **Comorbidities** |  |  |  |  |  |
| Arterial hypertension | 1,351 | 688 (50.9%) | 229 (26.5%) | 459 (94.4%) | <0.001 |
| Diabetes | 1,346 | 260 (19.3%) | 115 (13.3%) | 145 (30.0%) | <0.001 |
| Chronic Kidney Failure | 1,345 | 106 (7.9%) | 52 (6.0%) | 54 (11.2%) | <0.001 |
| COPD | 1,346 | 88 (6.5%) | 53 (6.1%) | 35 (7.2%) | 0.43 |
| Long-term oxygen therapy | 1,346 | 25 (1.9%) | 11 (1.3%) | 14 (2.9%) | 0.034 |
| Active solid neoplasm | 1,344 | 58 (4.3%) | 40 (4.6%) | 18 (3.7%) | 0.43 |
| Active hematologic malignancy | 1,345 | 53 (3.9%) | 41 (4.8%) | 12 (2.5%) | 0.041 |
| Cerebrovascular disease | 1,344 | 77 (5.7%) | 44 (5.1%) | 33 (6.8%) | 0.19 |
| Previous Myocardial Infarction | 1,346 | 130 (9.7%) | 57 (6.6%) | 73 (15.1%) | <0.001 |
| Chronic heart failure | 1,347 | 62 (4.6%) | 28 (3.2%) | 34 (7.0%) | 0.001 |
| Angina/previous revascularization | 1,316 | 143 (10.9%) | 64 (7.7%) | 79 (16.5%) | <0.001 |
| Atrial fibrillation | 1,320 | 129 (9.8%) | 67 (8.0%) | 62 (12.8%) | 0.004 |
| Vasculopathy | 1,349 | 119 (8.8%) | 58 (6.7%) | 61 (12.6%) | <0.001 |
| Rheumatic pathology | 1,344 | 67 (5.0%) | 39 (4.5%) | 28 (5.8%) | 0.30 |
| Immunosuppression | 1,345 | 80 (5.9%) | 55 (6.4%) | 25 (5.2%) | 0.37 |
| **Home therapies** |  |  |  |  |  |
| MRAs | 1,289 | 57 (4.4%) | 31 (3.8%) | 26 (5.5%) | 0.15 |
| Loop diuretics | 1,289 | 165 (12.8%) | 79 (9.7%) | 86 (18.2%) | <0.001 |
| Other diuretics | 1,287 | 138 (10.7%) | 16 (2.0%) | 122 (25.8%) | <0.001 |
| Beta-blockers | 1,287 | 324 (25.2%) | 140 (17.2%) | 184 (38.9%) | <0.001 |
| Calcium channel blockers | 1,352 | 356 (26.3%) | 153 (17.7%) | 203 (41.8%) | <0.001 |
| Statins | 1,288 | 315 (24.5%) | 127 (15.6%) | 188 (39.7%) | <0.001 |
| Steroids | 1,341 | 69 (5.1%) | 45 (5.2%) | 24 (5.0%) | 0.84 |
| Oral antidiabetics | 1,343 | 187 (13.9%) | 80 (9.3%) | 107 (22.2%) | <0.001 |
| Insulin | 1,343 | 73 (5.4%) | 38 (4.4%) | 35 (7.2%) | 0.028 |
| OAT/DOACs | 1,341 | 149 (11.1%) | 74 (8.6%) | 75 (15.5%) | <0.001 |
| Antiplatelets | 1,342 | 342 (25.5%) | 163 (19.0%) | 179 (37.0%) | <0.001 |
| Proton Pump inhibitors | 1,342 | 365 (27.2%) | 179 (20.8%) | 186 (38.6%) | <0.001 |
| **Symptoms on admission** |  |  |  |  |  |
| Fever | 1,335 | 1,172 (87.8%) | 755 (88.3%) | 417 (86.9%) | 0.44 |
| Cough | 1,334 | 590 (44.2%) | 409 (47.9%) | 181 (37.7%) | <0.001 |
| Anorexia | 1,333 | 108 (8.1%) | 73 (8.5%) | 35 (7.3%) | 0.43 |
| Asthenia | 1,334 | 354 (26.5%) | 227 (26.6%) | 127 (26.5%) | 0.96 |
| Myalgia | 1,334 | 81 (6.1%) | 54 (6.3%) | 27 (5.6%) | 0.61 |
| Dyspnoea | 1,334 | 847 (63.5%) | 541 (63.3%) | 306 (63.7%) | 0.88 |
| Sore throat | 1,333 | 27 (2.0%) | 19 (2.2%) | 8 (1.7%) | 0.55 |
| Dizziness | 1,333 | 63 (4.7%) | 42 (4.9%) | 21 (4.4%) | 0.65 |
| Abdominal pain | 1,334 | 35 (2.6%) | 23 (2.7%) | 12 (2.5%) | 0.83 |
| Diarrhoea | 1,333 | 121 (9.1%) | 77 (9.0%) | 44 (9.2%) | 0.93 |
| Nausea | 1,331 | 66 (5.0%) | 38 (4.5%) | 28 (5.8%) | 0.26 |
| Vomiting | 1,334 | 65 (4.9%) | 38 (4.4%) | 27 (5.6%) | 0.34 |
| Chest pain | 1,334 | 46 (3.4%) | 28 (3.3%) | 18 (3.8%) | 0.65 |
| Hypo/anosmia | 1,314 | 16 (1.2%) | 7 (0.8%) | 9 (1.9%) | 0.12 |
| Hypo/agenusia | 1,315 | 27 (2.1%) | 14 (1.7%) | 13 (2.7%) | 0.23 |
| **Vital signs at entry** |  |  |  |  |  |
| Heart Beat Frequency (bpm) | 1,180 | 84.5 (75.0-95.0) | 85.0 (76.0-95.0) | 84.0 (73.0-94.0) | 0.062 |
| Systolic blood pressure | 1,168 | 127.0 (115.0-140.0) | 125.5 (114.0-140.0) | 128.0 (115.0-145.0) | 0.057 |

**Table S3.** Characteristics of the 688 hypertensive Covid-19 patients stratifying by RASIs-use vs no RASIs-use. Symbol N stands for group numerosity. Symbol p stands for p-value for the difference between RASIs-use and no-RASIs-use populations with respect to a specific characteristic. For each yes-no characteristic (eg., male gender) the table reports number and percentage of "yes" patients within a particular stratum.

|  | **N non missing** | **Total** | **RASIs use** | | **p** | **No RASIs use** | | **p** |
| --- | --- | --- | --- | --- | --- | --- | --- | --- |
|  |  | **N=688** | **< 68 years**  **N=171** | **≥ 68 years**  **N=288** |  | **< 68 years**  **N=85** | **≥ 68 years**  **N=144** |  |
| Male gender | 688 | 489 (71.1%) | 139 (81.3%) | 196 (68.1%) | 0.002 | 57 (67.1%) | 97 (67.4%) | 0.96 |
| Age, *median (IQR)* | 688 | 72.0 (63.0-79.0) | 60.0 (55.0-64.0) | 77.0 (73.0-81.0) | <0.001 | 61.0 (56.0-65.0) | 78.5 (73.0-83.0) | <0.001 |
| BMI, *median (IQR)* | 503 | 27.3 (24.6-30.7) | 27.8 (25.0-32.4) | 26.9 (24.4-30.3) | 0.016 | 27.7 (25.4-31.2) | 26.5 (24.1-29.4) | 0.039 |
| Obesity (BMI > 30) | 512 | 149 (29.1%) | 47 (35.6%) | 59 (28.1%) | 0.14 | 22 (31.4%) | 21 (21.0%) | 0.12 |
| **Smoking history** |  |  |  |  |  |  |  |  |
| Current smoker | 606 | 20 (3.3%) | 4 (2.6%) | 6 (2.3%) | 0.58 | 5 (6.6%) | 5 (4.3%) | 0.75 |
| Former smoker |  | 144 (23.8%) | 30 (19.7%) | 63 (24.1%) |  | 19 (25.0%) | 32 (27.4%) |  |
| Never smoker |  | 442 (72.9%) | 118 (77.6%) | 192 (73.6%) |  | 52 (68.4%) | 80 (68.4%) |  |
| **Comorbidities** |  |  |  |  |  |  |  |  |
| Diabetes | 686 | 193 (28.1%) | 39 (23.1%) | 100 (34.7%) | 0.009 | 20 (23.5%) | 34 (23.6%) | 0.99 |
| CKF | 685 | 84 (12.3%) | 12 (7.1%) | 39 (13.6%) | 0.034 | 6 (7.1%) | 27 (18.8%) | 0.015 |
| COPD | 685 | 62 (9.1%) | 8 (4.7%) | 26 (9.1%) | 0.089 | 6 (7.1%) | 22 (15.3%) | 0.067 |
| Long-term oxygen therapy | 685 | 18 (2.6%) | 4 (2.4%) | 9 (3.1%) | 0.63 | 2 (2.4%) | 3 (2.1%) | 0.89 |
| Active solid neoplasm | 684 | 29 (4.2%) | 8 (4.7%) | 10 (3.5%) | 0.51 | 3 (3.5%) | 8 (5.6%) | 0.49 |
| Active hematologic malignancy | 684 | 24 (3.5%) | 6 (3.6%) | 6 (2.1%) | 0.37 | 3 (3.5%) | 9 (6.3%) | 0.54 |
| Cerebrovascular disease | 684 | 54 (7.9%) | 10 (5.9%) | 21 (7.3%) | 0.57 | 5 (5.9%) | 18 (12.6%) | 0.10 |
| Previous Myocardial Infarction | 683 | 101 (14.8%) | 19 (11.2%) | 49 (17.1%) | 0.089 | 6 (7.1%) | 27 (18.9%) | 0.014 |
| Chronic heart failure | 686 | 47 (6.9%) | 6 (3.5%) | 22 (7.7%) | 0.075 | 3 (3.5%) | 16 (11.1%) | 0.044 |
| Angina/previous revascularization | 671 | 109 (16.2%) | 17 (10.1%) | 55 (19.3%) | 0.009 | 9 (10.7%) | 28 (21.1%) | 0.049 |
| Atrial fibrillation | 674 | 93 (13.8%) | 10 (5.9%) | 50 (17.4%) | <0.001 | 4 (4.8%) | 29 (21.6%) | <0.001 |
| Vasculopathy | 686 | 91 (13.3%) | 13 (7.6%) | 48 (16.7%) | 0.006 | 8 (9.4%) | 22 (15.3%) | 0.20 |
| Rheumatic pathology | 685 | 38 (5.5%) | 6 (3.6%) | 20 (7.0%) | 0.13 | 2 (2.4%) | 10 (6.9%) | 0.13 |
| Immunosuppression | 684 | 39 (5.7%) | 12 (7.1%) | 12 (4.2%) | 0.18 | 8 (9.5%) | 7 (4.9%) | 0.17 |
| **Home therapies** |  |  |  |  |  |  |  |  |
| MRAs | 652 | 44 (6.7%) | 6 (3.7%) | 16 (5.6%) | 0.35 | 4 (5.0%) | 18 (14.5%) | 0.032 |
| Loop diuretics | 652 | 135 (20.7%) | 19 (11.6%) | 60 (21.1%) | 0.011 | 9 (11.3%) | 47 (37.9%) | <0.001 |
| Other diuretics | 651 | 132 (20.3%) | 41 (25.2%) | 77 (27.1%) | 0.65 | 5 (6.3%) | 9 (7.3%) | 0.78 |
| Beta-blockers | 650 | 266 (40.9%) | 48 (29.3%) | 125 (44.0%) | 0.002 | 31 (39.2%) | 62 (50.4%) | 0.12 |
| Calcium channel blockers | 688 | 332 (48.3%) | 69 (40.4%) | 129 (44.8%) | 0.35 | 50 (58.8%) | 84 (58.3%) | 0.94 |
| Statins | 651 | 242 (37.2%) | 44 (26.8%) | 135 (47.5%) | <0.001 | 20 (25.0%) | 43 (35.0%) | 0.13 |
| Steroids | 681 | 35 (5.1%) | 6 (3.6%) | 16 (5.6%) | 0.33 | 4 (4.8%) | 9 (6.3%) | 0.62 |
| Oral antidiabetics | 682 | 141 (20.7%) | 32 (18.9%) | 71 (24.7%) | 0.15 | 16 (19.0%) | 22 (15.5%) | 0.49 |
| Insulin | 682 | 51 (7.5%) | 10 (5.9%) | 24 (8.4%) | 0.34 | 4 (4.8%) | 13 (9.2%) | 0.23 |
| OAT/DOACs | 683 | 112 (16.4%) | 12 (7.1%) | 58 (20.1%) | <0.001 | 7 (8.3%) | 35 (24.6%) | 0.002 |
| Antiplatelets | 683 | 254 (37.2%) | 36 (21.3%) | 131 (45.5%) | <0.001 | 16 (19.0%) | 71 (50.0%) | <0.001 |
| Proton Pump inhibitors | 681 | 263 (38.6%) | 44 (26.3%) | 131 (45.5%) | <0.001 | 21 (25.0%) | 67 (47.2%) | <0.001 |
| **Symptoms on admission** |  |  |  |  |  |  |  |  |
| Fever | 679 | 581 (85.6%) | 149 (88.7%) | 243 (85.3%) | 0.30 | 74 (88.1%) | 115 (81.0%) | 0.16 |
| Cough | 678 | 264 (38.9%) | 81 (48.2%) | 89 (31.2%) | <0.001 | 43 (51.2%) | 51 (36.2%) | 0.027 |
| Anorexia | 678 | 48 (7.1%) | 12 (7.1%) | 21 (7.4%) | 0.93 | 4 (4.8%) | 11 (7.8%) | 0.38 |
| Asthenia | 678 | 187 (27.6%) | 36 (21.4%) | 85 (29.8%) | 0.051 | 22 (26.2%) | 44 (31.2%) | 0.42 |
| Myalgia | 678 | 39 (5.8%) | 12 (7.1%) | 12 (4.2%) | 0.18 | 7 (8.3%) | 8 (5.7%) | 0.44 |
| Dyspnoea | 678 | 431 (63.6%) | 113 (67.3%) | 177 (62.1%) | 0.27 | 58 (69.0%) | 83 (58.9%) | 0.13 |
| Sore throat | 677 | 11 (1.6%) | 3 (1.8%) | 4 (1.4%) | 0.71 | 3 (3.6%) | 1 (0.7%) | 0.15 |
| Dizziness | 678 | 26 (3.8%) | 4 (2.4%) | 15 (5.3%) | 0.14 | 3 (3.6%) | 4 (2.8%) | 0.76 |
| Abdominal pain | 678 | 16 (2.4%) | 2 (1.2%) | 8 (2.8%) | 0.26 | 5 (6.0%) | 1 (0.7%) | 0.018 |
| Diarrhoea | 678 | 62 (9.1%) | 13 (7.7%) | 30 (10.5%) | 0.33 | 8 (9.5%) | 11 (7.8%) | 0.65 |
| Nausea | 677 | 36 (5.3%) | 7 (4.2%) | 19 (6.7%) | 0.28 | 6 (7.1%) | 4 (2.8%) | 0.13 |
| Vomiting | 678 | 34 (5.0%) | 6 (3.6%) | 18 (6.3%) | 0.21 | 4 (4.8%) | 6 (4.3%) | 0.86 |
| Chest pain | 678 | 27 (4.0%) | 7 (4.2%) | 11 (3.9%) | 0.87 | 3 (3.6%) | 6 (4.3%) | 0.80 |
| Hypo/anosmia | 668 | 8 (1.2%) | 6 (3.6%) | 2 (0.7%) | 0.057 | 0 (0.0%) | 0 (0.0%) |  |
| Hypo/agenusia | 669 | 12 (1.8%) | 7 (4.1%) | 5 (1.8%) | 0.14 | 0 (0.0%) | 0 (0.0%) |  |
| **Vital signs at entry** |  |  |  |  |  |  |  |  |
| Heart Beat Frequency (bpm) | 604 | 83.0 (73.0-93.0) | 87.5 (78.0-97.0) | 80.0 (71.0-90.0) | <0.001 | 83.5 (74.0-93.0) | 80.0 (73.0-89.0) | 0.23 |
| Systolic blood pressure (mmHg) | 593 | 127.0 (113.0-142.0) | 126.0 (116.0-145.0) | 130.0 (115.0-145.0) | 0.44 | 127.0 (112.0-140.0) | 122.0 (110.0-140.0) | 0.75 |
| **Outcome** |  |  |  |  |  |  |  |  |
| Death | 688 | 240 (34.9%) | 34 (19.9%) | 121 (42.0%) | <0.001 | 12 (14.1%) | 73 (50.7%) | <0.001 |

BMI: Body mass index

CKF: Chronic kidney failure, defined as glomerular filtration rate <60 ml/min/m^2^.

COPD: chronic obstructive pulmonary disease

MRAs: mineralocorticoid receptor antagonists

OAT/DOACs: oral anticoagulant therapy/direct oral anticoagulants

***Diagnostic Analyses***

This section provides the Reader, with more insight into the part of our analysis that concerns the comparison between prior ARBs-users and no-RASIs-users. It looks into possible differences between these two exposure groups in terms of post-hospitalization variables.

TABLE S2 does not reveal marked differences between two matched ARBs and no-RASIs groups (120 patients each) in terms of clinical observations on hospital admission. These are the two groups used to assess the effect of ARBs in the population of 68+ hypertensives (the omitted variables showed less important discrepancies).

Nor do the same two groups markedly differ in terms of biochemical parameters measured upon hospital admission, according to Table S3. (The omitted parameters showed even smaller standardized discrepancies).

Similarly, the same two groups did also not markedly differ in terms of age, comorbidities and chronic therapies, according to Table S4.

**Table S4**. This table compares the ARBs/noACEIs-exposed group of 120 matched patients with the corresponding group of 120 matched RASIs-free patients (68+ hypertensives) in terms of some clinical variables measured upon hospital admission (those variables that revealed greater standardized discrepancies were chosen). There are no marked differences.

|  |  |  |  |  | Respiratory |  |
| --- | --- | --- | --- | --- | --- | --- |
| Exposure | n | Fever | Cough | Dispnea | Insufficiency |  |
| RASIs free | 136 | 105 | 41 | 78 | 104 |  |
| ARBs | 136 | 120 | 40 | 84 | 106 |  |
|  |  |  |  |  | Respiratory | Bilateral |
|  | n | Chest X Rays abnormalities | *pO*_2_ | *pCO*_2_ | *FiO*_2_*max*100 | P/F |
| RASIs free | 136 | 107 | 71.7 | 31.9 | 0.46 | 192 |
| ARBs | 136 | 95 | 76.4 | 32.3 | 0.44 | 206 |

**Table S5**. This table compares the same groups of the previous table in terms of some biochemical parameters measured upon hospital admission (those parameters that revealed greater standardized discrepancies were chosen). There were no remarkable differences.

| Exposure |  | n | LDH | AST | ALT | BILI | CREA | WBC | LYMPHco |
| --- | --- | --- | --- | --- | --- | --- | --- | --- | --- |
| RASIs-free |  | 136 | 467.79 | 64.41 | 45.66 | 0.73 | 1.16 | 8627 | 828 |
| ARBs |  | 136 | 520.57 | 93.97 | 72.84 | 0.72 | 1.62 | 8476 | 883 |
|  |  | n | Lymph% | NEUTco | NEUT% | PLT | PCR | UREA | HB |
| RASIs-free |  | 136 | 12.19 | 7121 | 82.1 | 216647 | 13.99 | 49.92 | 12.72 |
| ARBs |  | 136 | 11.89 | 7411 | 81.4 | 235448 | 12.72 | 58.22 | 12.54 |

**Table S6**: This table compares propensity-matched exposure groups (ARBs/no-ACEIs vs RASIs-free) in terms of comorbidity frequencies, frequencies of home therapies and average age.

|  |  | Mean |  |  | Cereb. |
| --- | --- | --- | --- | --- | --- |
|  |  | num of | Loop | Other | vasc. |
| Exposure | n | comorbid. | diuretics | diuretics | path. |
| RASIs-free | 136 | 0.86 | 31 | 37 | 7 |
| ARBs/no ACEIs | 136 | 0.83 | 27 | 47 | 9 |

| ARBs | Statins | Insulin | Inhalers | COPD | Age |
| --- | --- | --- | --- | --- | --- |
| no | 63 | 10 | 9 | 24 | 78.0 |
| yes | 65 | 13 | 9 | 22 | 78.0 |

**Table S7**: estimated propensity score for chronic use of ARBs in population of 68+ Covid-19 patients with hypertension (ACEIs users excluded). This particular score is defined as the logarithm of the odds of probability of the generic 68+ hypertensive patient having been exposed to ARBs prior to hospitalization, conditional on their medical history and on not having used ACEIs. The score for a generic patient is obtained by summing up the contributions from each row of the table. For example, if the patient uses “Other Anti-hypertensives”, this will contribute into the score the quantity -0.29 (estimated). Their age will contribute a quantity equal to -0.037 times the number of years of age, and so on. Note that calculation of the score requires that none of the listed information items is missing.

|  |  | Std. |  |
| --- | --- | --- | --- |
|  | Est. | Err. | *p* |
| (Intercept) | 2.11 | 2.16 | 0.33 |
| Other anti-hypertensives | -0.29 | 0.32 | 0.36 |
| Beta-blockers | 0.11 | 0.32 | 0.72 |
| Loop Diuretics | -0.84 | 0.45 | 0.06 |
| Other diuretics | 2.51 | 0.47 | .000 |
| Statins | 0.89 | 0.37 | .016 |
| Number of comorbidities | 1.11 | 0.86 | 0.19 |
| Cerebrovascular pathology | -2.22 | 1.07 | .039 |
| Age (yrs) | -.037 | .028 | .186 |
| Male gender | -0.02 | 0.33 | 0.94 |
| Previous acute myocardial infarction | -0.94 | 1.00 | 0.34 |
| Chronic Heart Failure | 0.22 | 0.62 | 0.72 |
| Diabetes Mellitus | 0.60 | .608 | 0.32 |
| Atrial fibrillation | -0.29 | 0.89 | 0.74 |
| Pre-Covid steroids | -0.63 | 0.79 | 0.42 |
| Oral anti-diabetic | 0.55 | 0.62 | 0.37 |
| Insulin | -1.78 | 1.08 | .099 |
| COPD Inhalers | 0.77 | 0.72 | 0.28 |
| Anti-platelet agents | -0.16 | 0.35 | 0.64 |
| Chronic Renal Failure | -0.52 | 0.48 | 0.27 |
| Chronic Obstructive Pulm. Disease | -0.34 | 0.61 | 0.57 |
| Proton pump inhibitors | 0.46 | 0.35 | 0.19 |
| Coronary Artery Disease | -1.22 | 0.59 | .038 |
| Liver Cirrhosis | -17.7 | 976 | 0.98 |
| Rheumatic pathology | -0.82 | 1.04 | 0.42 |
| Peptic Ulcer | -16.7 | 1144 | 0.98 |
| Vasculopathy | -0.32 | 0.96 | 0.73 |

**Extended list of authors including members of HPG23 Covid Lab:**

Mauro Gori MD, Carlo Berzuini PhD, Emilia D’Elia MD PhD, Arianna Ghirardi PhD, Luisa Bernardinelli MD PhD, Antonello Gavazzi MD, Giulio Balestrieri MD, Andrea Giammarresi MD, Trevisan Roberto MD, Fabiano Di Marco MD PhD, Antonio Bellasi MD, Mariangela Amoroso MD, Federico Raimondi MD, Luca Novelli MD, Bianca Magro MD, Gianpaolo Mangia MD, Ferdinando Luca Lorini MD, Giulio Guagliumi MD, Stefano Fagiuoli MD, Gianfranco Parati MD, Michele Senni MD, Marco Rizzi MD, Elena Gervasi MD, Binda Francesca MD, Arianna Masciulli MD, Tiziano Barbui MD, Alessandro Rambaldi MD, Roberto Cosentini MD, Lorenzo Stephan Cesare Grazioli MD, Gianmariano Marchesi MD, Francesco Ferri MD, Piercarlo Parigi MD, Caterina Conti MD, Roberta Civiletti MD, Roberta Biza MD, Roberta Trapasso MD, Lisa Giuliani MD, Marisa Anelli MD, Chiara Allegri MD, Gianluca Imeri MD, Claudia Sanfilippo MD, Sofia Comandini MD, England Hila MD, Leonardo Manesso MD, Lucia Gandini MD, Pietro Mandelli MD, Martina Monti MD, Dario Pellegrini MD, Stefania Camagni MD, Amaduzzi Annalisa MD, Luca Del Prete MD, Michele Colledan MD, Lorenzo D’Antiga MD, Luisa Pasulo MD, Andrea Gianatti MD, Claudio Farina MD, Annapaola Callegaro MD, Sabrina Buoro MD, Sandro Sironi MD, Piero Ruggenenti MD, Valentina Portalupi MD, Camillo Carrara MD, Laura Cappelletti MD, Anna Falanga MD, Luigi Filippo Da Pozzo MD, Maria Sessa MD, Maria Grimoldi MD, Giorgia Camera MD, Fabio Pezzoli MD, Simonetta Cesa, Monica Casati

**Affiliations of co-authors:** From the **Infectious Diseases Unit** (M.R., E.G., F.B.), From **Research Foundation of Bergamo Hospital**  (A.M., T.B.), the **Department of Oncology and Hematology** (A.R.), the **Emergency Department** (R.C.), the **Intensive Care Unit** (L.S.C.G., G.M., F.F.,), the **Unit of Pulmonary Medicine** (P.P., C.C., R.C., M.A., R.B., R.T., L.G., M.A., C.A., G.I., C.S., S.C.), **Cardiovascular Department** (D.P.), the **Department of Organ Failure and Transplantation** (S.C., A.A., L.D.P., M.C.), the **Paediatric Hepatology Gastroenterology and Transplantation Unit** (L.D.A.), the **Gastroenterology Hepatology and Transplantation Unit** (L.P.), the **Pathology Unit Department of Laboratory Medicine** (A.G.), the **Department of Laboratory Medicine** (C.F., A.C.), the **Quality Management Unit** (S.B.), the **Department of Diagnostic Radiology** (S.S.), the **Nephrology Unit** (P.R., V.P, C.C., L.C.), the **Immunohematology and Transfusion** (A.F.), the **Urology Unit** (L.F.D.P), the **Neurology Unit** (M.S., M.G., G.C.), the **Medical Direction** (F.P.), the **Department of Health and Social Care Professions** (S.C.), the **Research, Education and Development Unit Department of Health and Social Care Professions** (M.C.), **ASST Papa Giovanni XXIII – Bergamo**. **University of Milan** (F.D.M., F.R., G.R., E.H., L.M., L.G., P.M., M.M.), Milan, **University of Milan-Bicocca** (A.F.), Milan - all in Italy.

**From the Centre for Biostatistics, The University of Manchester, Manchester, UK** (C.B.), **the Department of Brain and Behavioural Sciences, The University of Pavia, Pavia, Italy** (L.B.).
